# Evaluating the immunogenicity of heparin and heparin derivatives by measuring their binding to platelet factor 4 using biolayer interferometry

**DOI:** 10.3389/fmolb.2022.966754

**Published:** 2022-08-26

**Authors:** Qingqing Chen, Fei Li, Haoran Wang, Changkai Bu, Feng Shi, Lan Jin, Qunye Zhang, Lianli Chi

**Affiliations:** ^1^ National Glycoengineering Research Center, Shandong University, Qingdao, China; ^2^ Scientific Research Division, Shandong Institute for Food and Drug Control, Jinan, China; ^3^ The Key Laboratory of Cardiovascular Remodeling and Function Research, Chinese Ministry of Education, Chinese National Health Commission and Chinese Academy of Medical Sciences, Qilu Hospital, Shandong University, Jinan, China

**Keywords:** heparin, platelet factor 4, interaction, molecular mechanism, biolayer interferometry

## Abstract

Heparin (HP) is a polysaccharide that is widely used in the clinic as an anticoagulant. A major side effect associated with HP is the heparin-induced thrombocytopenia (HIT), which is initiated by the immune response to complex formed by HP and platelet factor 4 (PF4). Low molecular weight heparins (LMWHs) are the depolymerized version of HP, which have reduced risks of inducing HIT. However, it is still necessary to evaluate the immunogenicity of LMWHs to ensure their drug safety. Since HIT involves very complicated processes, the evaluation of HP and LMWH immunogenicity requires experiments from multiple aspects, of which the binding affinity between HP and PF4 is a key property to be monitored. Herein, we developed a novel competitive biolayer interferometry (BLI) method to investigate the binding affinity between HP and PF4. The influence of different domains in HP on its immunogenicity was compared for better understanding of the molecular mechanism of HP immunogenicity. Furthermore, the half maximal inhibitory concentration (IC_50_) of HP and LMWH can be measured by competitive combination, which is important for the quality control during the developing and manufacturing of HP and LMWH drugs.

## Introduction

**FIGURE 1:**
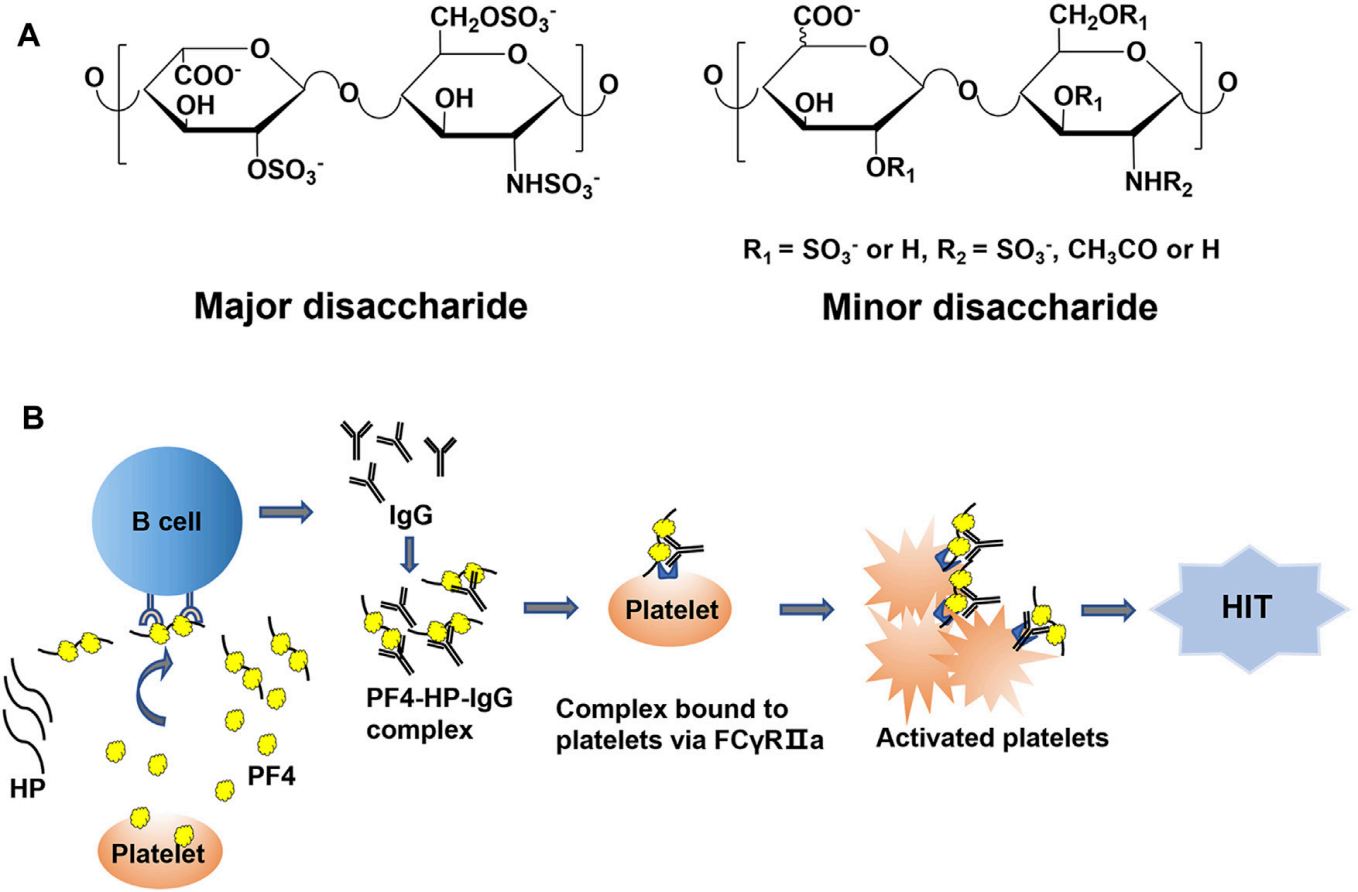
**(A)** The structure of HP. **(B)** The pathophysiologic process of HIT.

Heparin (HP), a polysaccharide belonging to the glycosaminoglycan (GAG) family, is comprised of iduronic residue (IdoA) or glucuronic acid residue (GlcA) alternating linked to glucosamine (GlcN) repeating disaccharide units with variable sulfation and/or acetylation substitutions (Figure 1A). The structural properties of HP allow it to interact with a variety of proteins *in vivo* and play a series of biological functions. The most important biological function of HP is anticoagulation. Due to its binding to antithrombin III and increasing the anticoagulant potential, HP is widely used in the prevention and treatment of thrombotic diseases (Capila and Linhardt, 2002; Kearon et al., 2012). However, HP is also capable of binding to other proteins, such as platelet factor 4 (PF4), putting it at risk of causing side effects, for example, the heparin-induced thrombocytopenia (HIT). To reduce the side effects of HP in clinical application, different versions of low molecular weight HPs (LMWHs) have been developed (Fareed et al., 1998; Xu et al., 2011; McGowan et al., 2016; Xu et al., 2017). The molecular weight (MW) of LMWHs is usually ranged from 3,000 to 8,000 Da, degraded from HP by chemical or enzymatic depolymerization (Linhardt and Liu, 2012). With the decrease of MW, the binding capacity of LMWHs to plasma proteins and vascular endothelial cells (ECs) drops significantly. However, the risk of causing HIT by LMWHs still exists (Spinler and Dager, 2003; Malik et al., 2021).

HIT is one of the most common and serious side effects during the HP and LMWH therapy, which can further induce hemorrhage, thrombosis, and even death (Ernest and Fisher, 1991; Warkentin et al., 2005). The incidence of HIT is about 0.2%–5% in patients treated with HP, which is classified as type I and type II. Type I HIT is non-immune-mediated thrombocytopenia, usually presenting only with mild thrombocytopenia, and compared with type II HIT, there is little risk of thrombosis. The pathogenesis of type II HIT is mainly related to the immune response mediated by PF4-HP complex. When platelets are activated by bacterial infection, surgical trauma and other stimulation factors, PF4 is released by activated platelets and may interact with HP or LMWHs to form PF4-HP or PF4-LMWH complex. The complex can serve as antigen epitopes to stimulate B cells to produce antibodies. Then these antibodies bind to the PF4-HP complex and form antigenic complex. The ultra-complex of PF4/HP/immunoglobulin (IgG) cluster promotes platelet activation and aggregation by binding to FcγRIIa (CD32a) on platelets, leading to increased platelet consumption, eventually presents with symptoms of thrombocytopenia (Figure 1B). If patients with HIT do not receive timely and effective treatment, it can lead to a series of life-threatening diseases, such as myocardial infarction, deep vein thrombosis and pulmonary embolism. The formation of PF4-HP complex is the major inducement for HIT (Greinacher et al., 2008). Therefore, it is of great need to develop a method to evaluate the binding potency between HP/LWMH products and PF4, which improves the understanding of the structural characteristics influencing the interaction.

Binding kinetics and affinity are main indicators to reflect the interaction of biological macromolecules. Techniques that are capable to measure binding kinetics and affinity include isothermal titration calorimetry, microscale thermophoresis, surface plasmon resonance (SPR), and biolayer-interferometry (BLI) (Kairys et al., 2019). Among these techniques, SPR and BLI are real-time, and of other advantages of fluorescent label-free, high sensitivity and high reproducibility (Concepcion et al., 2009; Nguyen H. H. et al., 2015). Zhang and coworkers have developed an SPR method to investigate the interaction between HP and PF4. The HIT potential of a series of heparin products were evaluated by determining the half maximal inhibitory concentration (IC_50_) of them to PF4 (Zhang et al., 2020). BLI is a technique that more recently developed after SPR. With different immobilization and detection mechanisms, BLI provides an alternative way to evaluate the binding behaviors of HP and HP derivatives to PF4.

In the present study, we developed a competitive BLI method to study the binding affinity of HP and its derivatives to PF4. The contributions of different substitutions on HP to the binding to PF4 were investigated. The immunogenicity of HP and LMWH were also evaluated, which offers a sensitive and robust tool for ensuring and improving the safety of HP-based anticoagulant drugs.

## Materials and methods

### Materials

HP and LMWH (enoxaparin) were obtained from the United States Pharmacopoeia convention. Recombinant human PF4 was purchased from Sino Biological (Beijing, China). NHS-LC-LC-Biotin was purchased from Thermo Fisher Scientific (Shanghai, China). Deuterium oxide (99.9% D_2_O) was obtained from Qingdao Tenglong Weibo Technology Co., Ltd. (Qingdao, China). The 70% aqueous hydrazine was obtained from Sinopharm Chemical Reagent Co., Ltd. (Shanghai, China). Hydrazine sulfate, dimethyl sulfoxide, pyridine and Dowex 50WX8 (hydrogen form, 50 to 100 mesh) were purchased from Sigma-Aldrich (Shanghai, China). *N*, *O*-bis(trimethylsilyl)acetamide (BTSA) was obtained from Jinming Biotechnology (Beijing, China). Phosphate-Buffered Saline (PBS) was purchased from Wuhan Procell Biological Technology (Wuhan, China).

## Methods

### Kinetic measurement of interaction between HP and PF4

HP was incubated with NHS-LC-LC-Biotin in the molar ratio of 1:1 at room temperature for 1 h. After incubation, the mixture was desalted with 3 kDa MW cut off (MWCO) ultra-centrifugal filters (Kinsella et al., 1998). Biotinylated HP was then immobilized onto the streptavidin (SA) sensors.

PF4, the molecular weight is 7.8 kDa, was diluted to five different concentrations in PBS buffer (1 µM, 0.75 µM, 0.5 µM, 0.25 µM, and 0.13 µM). The BLI assay included the following steps: baseline 1 (PBS buffer), loading (biotinylated HP), baseline 2 (PBS buffer), association (PF4 samples), and dissociation (PBS buffer). The association and dissociation curves were acquired with the plate temperature at 30°C and the acquisition rate as standard kinetics (5.0 Hz, averaging by 20). ForteBio Data Analysis 10.0 software was used for data processing. The experimental data were fit with the 1:1 Langmuir model, and analyzed with global fitting to calculate association rate constant (*k*_on_), dissociation rate constant (*k*_dis_) and dissociation constant (K_D_ = *k*_dis_/*k*_on_).

### Preparation of HP derivatives

A variety of HP derivatives were prepared by selective removal of sulfo or acetyl substitution groups. The *N*-deacetylated HP was prepared according to the previously described protocol (Guo and Conrad, 1989). Briefly, 5 mg of HP was added to the mixture of 70% aqueous hydrazine (8 ml) and hydrazine sulfate (80 mg). The reaction mixture was stirred at 97°C for 4 h and then was quenched with ice water bath.

The 2-*O*-desulfated HP was prepared by sodium hydroxide treatment (Jaseja et al., 1989). The 0.1 M sodium hydroxide was added to the HP solution (6 mg in 1.5 ml distilled water) to reach a final pH of 12.5–12.8. After reaction, the solution was lyophilized and neutralized with 0.1 M hydrochloric acid.

The 6-*O*-desulfated HP was prepared by using BTSA to treat the HP pyridinium salt (Matsuo et al., 1993). HP was changed to its pyridinium salt by neutralizing with pyridine after passing a column packed with Dowex 50WX8 resin. The pyridinium salt was redissolved in pyridine. BTSA (1:10, v:v) was added, and the reaction was carried out at 60°C for 2 h. The 0.1 M sodium hydroxide was used to adjust the pH to 7.5 after impurities are removed by dialysis.

The *N*-desulfated HP was prepared by using dimethyl sulfoxide and H_2_O to treat the HP pyridinium salt (Nagasawa et al., 1977). The pyridinium salt of HP was dissolved in a mixture of dimethyl sulfoxide and H_2_O (19:1, v:v) and kept at 55°C for 1.5 h. The 0.1 M sodium hydroxide was used to adjust the pH to 9.0. The structures of all HP derivatives were confirmed by ^1^H-nuclear magnetic resonance (NMR) and ^1^H-^13^C heteronuclear single quantum coherence (HSQC)-NMR.

### Evaluation of the impact of HP substitutions on PF4-HP interaction

HP derivatives were mixed with PF4 at the concentrations of each HP derivative, 0.25 μg/ml, and PF4 0.5 µM. The BLI assay included the following steps: baseline 1 (PBS buffer), loading (biotinylated HP), baseline 2 (PBS buffer), association (HP derivative and PF4), and dissociation (PBS buffer). The parameters of instrument and data processing were same as above.

### Molecular docking and modeling simulation

Molecular docking and modeling of the PF4 with HP dodecasaccharide, different desulfated HP dodecasaccharides were performed using AutoDock 4.2.6 software. The structure of HP dodecasaccharide was retrieved from the Protein Data Bank (PDB) under code 1HPN, and a crystal structure was used for PF4 tetramer (PDB: 1F9Q). The structures of desulfated HP dodecasaccharides were edited and optimized on the basis of HP dodecasaccharides. All of the hydrogen atoms were added to PF4 and Gasteiger charge was used. A volume of (126, 126, 126) grid points with 0.519 Å spacing was built. PF4 tetramer was placed in the center of this box to completely cover the surface. Dodecasaccharides were put casually in the box. During the docking process, all monosaccharide rings were fixed at their starting conformations, while the sulfo groups were defined as flexible and could be rotated freely. The Lamarckian GA (4.2) was used for docking.

### Competition IC_50_ measurement for HP and LMWH

PF4 (0.5 µM) samples were mixed with different concentrations of HP or LMWH in PBS buffer. The concentrations of HP were 1 μg/ml, 0.75 μg/ml, 0.5 μg/ml, 0.25 μg/ml, and 0.13 μg/ml, he molar ratio of HP to PF4 was 0.13:1, 0.094:1, 0.063:1, 0.031:1, and 0.016:1. The concentrations of LMWH were 7 µg/ml, 5 µg/ml, 2, 1 µg/ml, and 0.5 μg/ml, and the molar ratio of LMWH to PF4 was 3.1:1, 2.2:1, 0.88:1, 0.44:1, and 0.22:1. The experimental process and instrument parameters were the same as above.

## Results

### Kinetic measurement of interaction between HP and PF4

**FIGURE 2:**
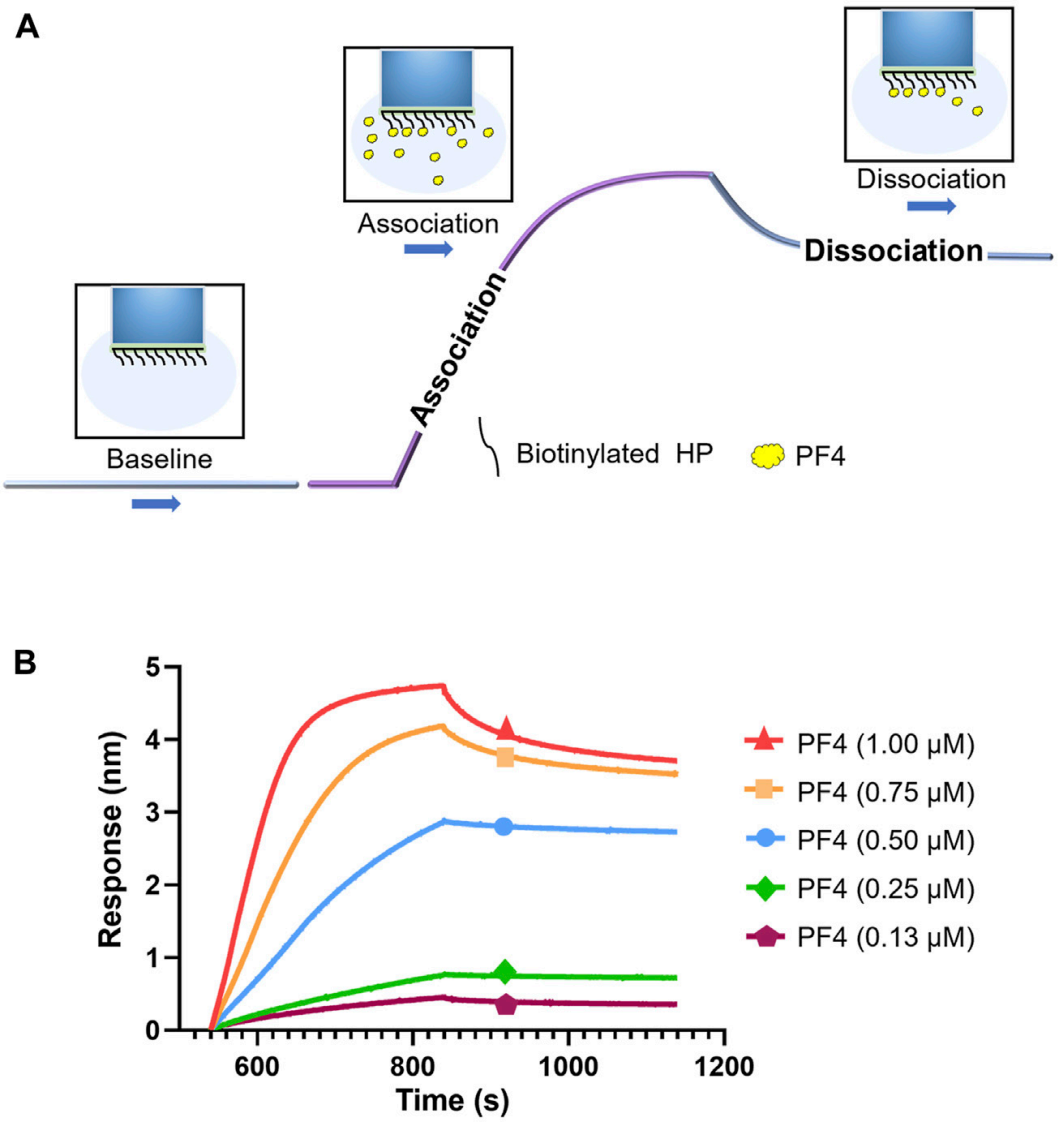
Kinetic measurement of interaction between HP and PF4. **(A)** Workflow of kinetic measurement using BLI. **(B)** BLI sensorgrams of PF4-HP interaction.

To measure the kinetic process of interaction between PF4 and HP, BLI method was used. The biotinylated HP were coupled on the surface of SA sensors to interact with different concentrations of PF4 in solution, which resulted in changes of the optical signal. The workflow of affinity assay is shown in Figure 2A. Sensorgram curves of the interaction between PF4 and HP were shown in Figure 2B. The high concentration curve reached equilibrium during the experiment, and the curves fit well to a 1:1 Langmuir biomolecular reaction model (R^2^ > 0.85). The *k*_on_, *k*_dis_, K_D_ were calculated, as *k*_on_ was 1.14 × 10^4^ (±1.32 × 10^2^) M^−1^s^−1^, *k*_dis_ was 6.29 × 10^–4^ (±1.44 × 10^–5^) s^−1^, and K_D_ for the PF4-HP interaction was 55.2 (±1.42) nM, respectively.

### Effects of different HP substitutions on the PF4-HP interaction

To evaluate how every type of substitution on HP effected the PF4-HP interaction, a variety of HP derivatives were prepared by selective removal of each *N*-sulfo group, *O*-sulfo group or *N*-acetyl group. The structure of each HP derivative was confirmed by ^1^H NMR and ^1^H-^13^C-HSQC NMR. The purity was greater than 80% based on the ^1^H NMR spectra (Supplementary Figure S1).

**FIGURE 3:**
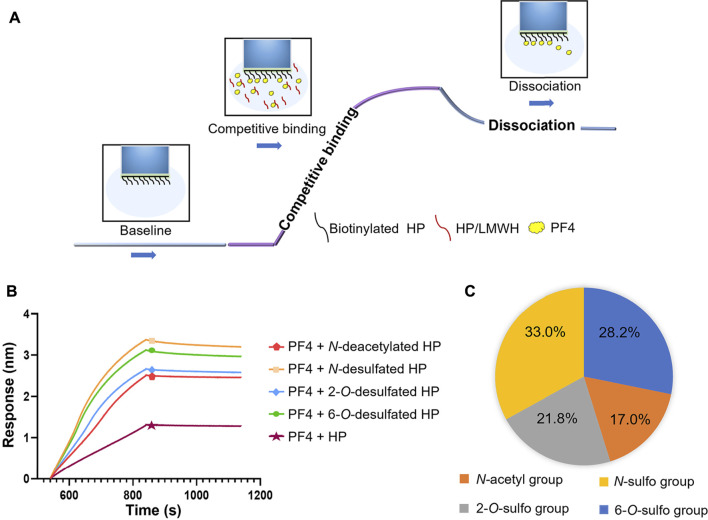
Effects of different HP substitutions on the PF4-HP interaction. **(A)** The diagram of competitive BLI method. **(B)** Competitive binding sensorgrams of HP and HP derivatives (*N*-deacetylated HP, *N*-desulfated HP, 2-*O*-desulfated HP, and 6-*O*-desulfated HP). **(C)** The contribution percentage of each substitution type to HP binding to PF4.

A competitive BLI method was then developed to evaluate the change of PF4 binding potentials corresponding to every substitution type on HP, by measuring the change of amount of PF4 bound to HP immobilized on the surface of sensors when the PF4 in solution was competitively bound by HP derivatives (Figure 3A). The signal strength compared to the control group reflected the contribution of each type of HP substitution to the binding between PF4 and HP. Sensorgrams of the competitive binding of HP derivatives to PF4 were shown in Figure 3B. As a positive control, the original HP showed the greatest potential to competitively bind to PF4, as its curve was of lowest signal strength. Compared to the starting HP, the curves of HP derivatives all changed significantly, but shifted to different extents. The order of contribution of each substitution group to bind to PF4, from greatest to least, was *N*-sulfo group, 6-*O*-sulfo group, 2-*O*-sulfo group, and *N*-acetyl group (Figure 3C).

### Molecular docking and modeling simulations for PF4 and HP

**FIGURE 4:**
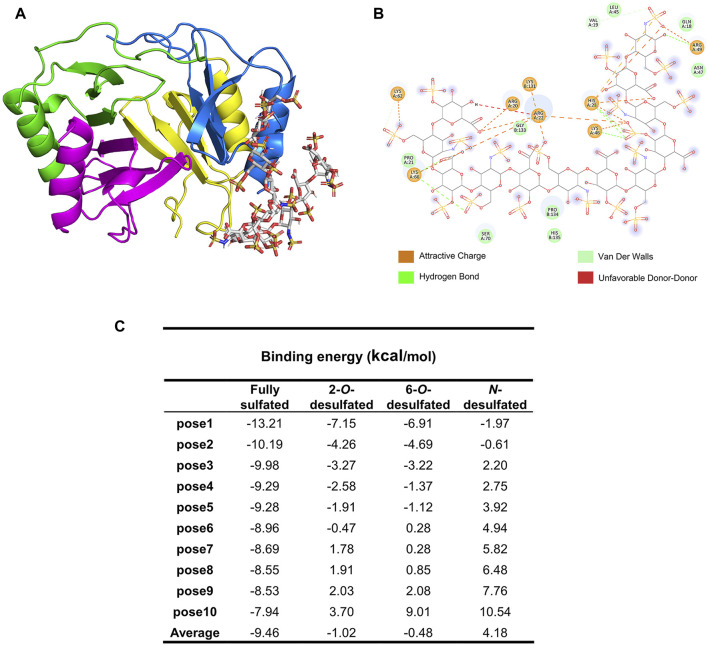
Molecular docking and modeling simulation of PF4 and HP dodecasaccharides. **(A)** The complex of the PF4 tetramer (PDB number: 1F9Q) and fully sulfated HP dodecasaccharide (PDB number: 1HPN) generated by AutoDock 4.2.6 and shown in Pymol. **(B)** The contributions of amino acid residues of PF4 and acidic groups of HP dodecasaccharide to the binding and the types of interactions involved in the binding. **(C)** Binding energy of different HP dodecasaccharides to PF4.

To demonstrate binding patterns and the contribution of each type of substitution on HP to its binding to PF4, theoretical binding models of these two biomolecules were constructed. The HP oligosaccharides with sizes of 12-mer were used to represent the binding domains of HP polysaccharide, while the tetramer of PF4 (consisting of monomer A, B, C and D) was chosen to represent the natural form of the protein. The complex of the PF4 tetramer and fully sulfated HP dodecasaccharide was visualized using the Pymol software and shown in Figure 4A. The amino acid residues on PF4 and negatively charged groups on HP involved in the interaction were shown in Figure 4B, using the optimal conformation pose 1 of the docking models of the PF4 tetramer and the fully sulfated dodecasaccharide. The binding between these two molecules was mainly controlled by electrostatic forces, while hydrogen bonds and Van der Waals forces also assisted to stabilize it. Specifically, the carboxyl groups interacted with the basic amino acid residues R20, R22, H23 and K66 of monomer A, the 2-*O*-sulfo group also interacted with R22, K46 and H23 residues, the 6-*O*-sulfo group interacted with the K62 residue on monomer A and K131 on monomer B, and the *N*-sulfo group at the reducing end interacted with the R49 residue of monomer A.

To mimic the HP derivatives, the sulfo groups were individually removed by editing the theoretical structure of fully sulfated HP dodecasaccharide. The binding energy of top 10 favored poses were calculated and presented in Figure 4C. The fully sulfated dodecasaccharide and PF4 had the lowest averaged binding energy, suggesting it was the strongest binding. In comparison, the averaged binding energies of HP derivatives increased, indicating that the binding became weaker if sulfo groups were removed. The order of binding energy increase was *N*-desulfated, 6-*O*-desulfated, and 2-*O*-desulfated dodecasaccharides, from high to low, which was consisted to the trend of BLI measurement.

### Competition IC_50_ measurement of HP and LMWH by BLI

**FIGURE 5:**
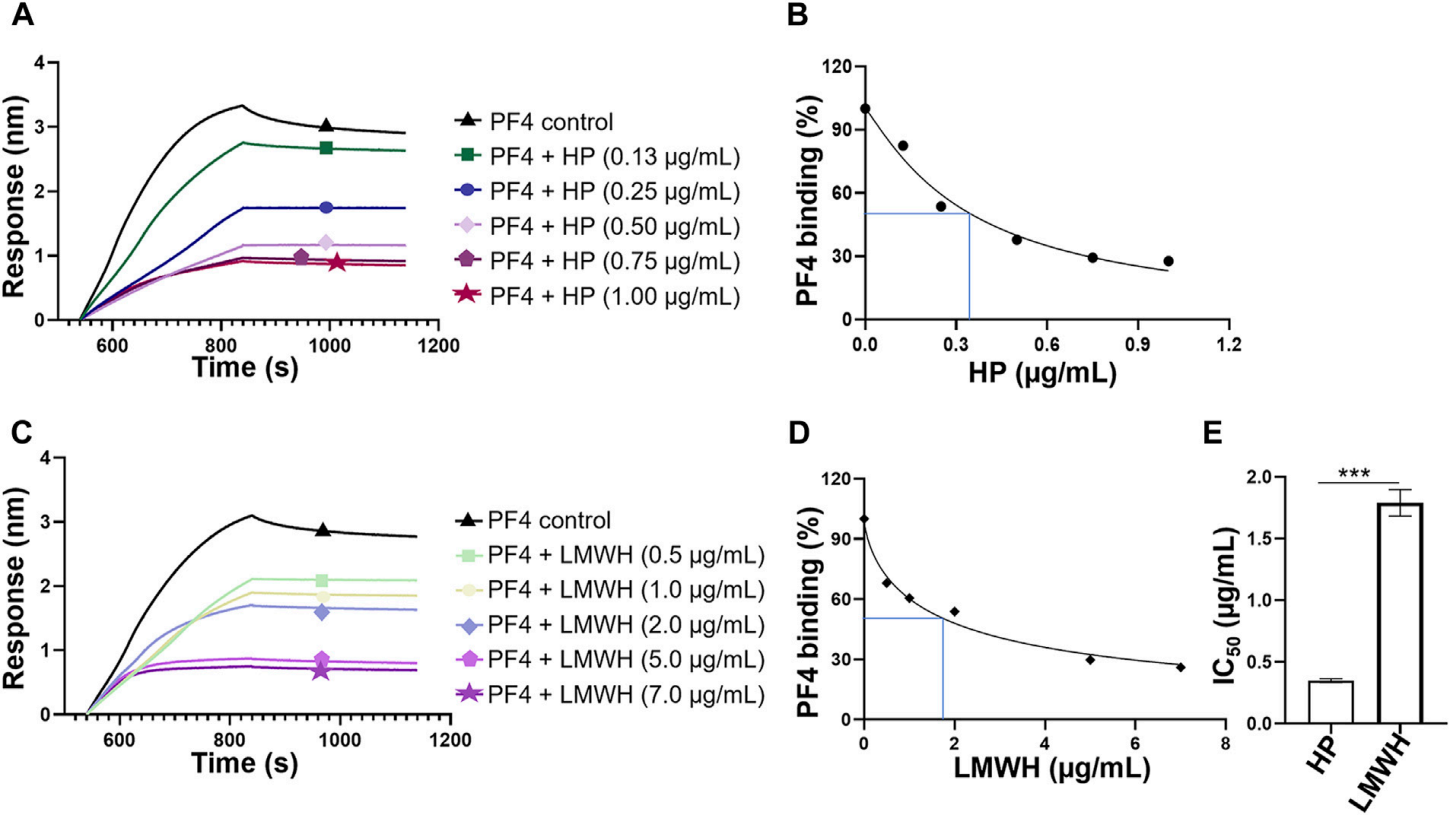
Competition IC_50_ measurement of HP and LMWH by BLI. **(A)** Competitive binding sensorgrams of PF4-HP interaction inhibiting by different concentrations of HP. The concentrations of HP were 1.00 μg/ml, 0.75 μg/ml, 0.5 μg/ml, 0.25 μg/ml, and 0.13 μg/ml. **(B)** Calculation of IC_50_ for HP. **(C)** Competitive binding sensorgrams of PF4-HP interaction inhibiting by different concentrations of LMWH. The concentrations of LMWH were 7 μg/ml, 5 μg/ml, 2 μg/ml, 1 μg/ml, and 0.5 μg/ml. **(D)** Calculation of IC_50_ for LMWH **(E)** Comparison of IC_50_ values between HP and LMWH.

Competitive binding to PF4 between surface immobilized HP and HP or LMWH in solution were performed to obtain their IC_50_ values. The sensorgrams showed that when HP or LMWH interacted to PF4 in solution, the PF4 bound to the surface-immobilized HP was reduced, reflected as the decrease of signal strength (Figures 5A,C). The IC_50_ values were calculated using the normalized PF4 binding signals and the concentration series of HP or LMWH in solution (Figures 5B,D). The IC_50_ values of HP and LMWH were 0.35 µg/ml and 1.79 μg/ml, respectively. The binding ability of HP to PF4 was significantly higher than that of LMWH (*p* < 0.001) (Figure 5E).

## Discussion

HP is a linear polysaccharide with high negative charge, which can bind to many positively charged amino acid residue clusters of proteins by electrostatic attraction, hydrogen bonding and Van der Waals force. This property of HP puts it at risk of triggering several side effects when being used as an anticoagulant. HIT is one immune system involved side effect that triggered by the undesired interaction between HP and PF4. Therefore, characterization of the binding behavior between HP and PF4 is of great importance. Since each individual method has its advantage and limitation, orthogonal methods are usually required to overcome these limitations and provide a completely assessment of the immunogenicity risk for HP and LMWH products. BLI is a relatively newly emerged technique for studying binding kinetics between biomacromolecules with high sensitivity and high precision, which is complementary to the classic SPR technology (Zhang et al., 2020). To avoid the hindering of assessing any major substitution type of HP, the unfrequently occurred unsubstituted amine group of HP was utilized to be immobilized on the BLI sensors (Sun et al., 2016). On the other hand, the PF4 protein could be in its natural conformation in solution during the interaction measurement. Thereof, the competitive BLI method developed herein could more closely reflect the PF4-HP interaction in the physiological condition. It also can be easily used to further study the possible molecular mechanism of interaction between PF4 and HP by testing a set of HP derivatives with distinct substitution patterns.

The repeating disaccharides construct the backbone of HP, while the substitution variability raises the structural complexity and heterogeneity of HP. The major substitution forms of HP include 2-*O*-sulfation at the IdoA and GlcA residue, and 6-*O*-sulfation, *N*-sulfation, *N*-acetylation at the GlcN residue. The 3-*O*-sulfation at the GlcN residue is rarely occurred, but is essential for the anticoagulant activity of HP. The variable sulfo groups of HP provide the high negative charge density, in addition to the carboxyl group uniformly located on every repeating disaccharide. When HP binds to certain protein, only part of the polysaccharide is involved in the interaction. For example, the binding domain to antithrombin III has been identified as a pentasaccharide with sequence of GlcN(Ac/S)6S-GlcA-GlcNS3S(6S/6OH)-IdoA2S-GlcNS6S, after capturing these antithrombin III binding fragments using affinity chromatography (Choay et al., 1981; Choay et al., 1983). In another case of specific GAG-protein binding, the 2-*O*-sulfo substitution was found to be crucial during heparan sulfate bound to high mobility group Box 1, and through the binding, heparan sulfate could neutralize this inflammatory factor and attenuate liver injury (Nam et al., 2017). Thereof, the different substitutions of HP may play different roles during the binding to PF4. To investigate it, the competitive BLI method was used to measure the binding between PF4 and HP derivatives produced by selective removal of each type of substitution. Compared to the intact HP, the binding affinity was weakened after any sulfo group or acetyl group was removed. However, each substitution affected the ability of HP binding to PF4 at different degrees. The *N*-acetyl group showed the least impact, likely due to the inability of it to provide the negative charge needed to interact with the positively charged domain of PF4. Three types of sulfo groups showed stronger influence on the binding. Within them, the *N*-sulfo group and 6-*O*-sulfo group were more important than the 2-*O*-sulfo group, suggesting the fine structure of HP may closely related to its immunogenicity.

Molecular docking further explained the role of each type of substitution during the PF4 binding and also predicted the amino acid residues on PF4 that participated in the interaction. The previous study by NMR and site-directed mutagenesis has revealed that R20, R22, H23, T25, K46 and R49 residues of PF4 significantly affected the PF4-HP binding (Mayo et al., 1995). The molecular docking result not only confirmed the significance of R20, R22, H23, K46 and R49 residues, but also indicated the involvement of K62, K66 and K131 residues in the PF4-HP binding. A more complete picture of the molecular basis of formation of PF4-HP complex was provided, which is fundamental for developing strategies to control the immunogenicity of HP and reducing the occurrence of HIT in future.

While ongoing efforts are being made to develop next-generation of anticoagulants with least immunogenicity, there is an urgent need of analytical method that can sensitively reflect the immunogenicity of HP and LMWH products currently used in clinic or being developed as generic drugs. The competition IC_50_ obtained by BLI is an indicator of the ability of HP or LMWH product to form complex with PF4 and further inducing immunogenicity. The BLI assay was capable to differentiate the immunogenicity potential of a HP product and a LMWH product. The IC50 value of LMWH was over 5-fold to that of HP when the concentration of PF4 is 0.5 µM, indicating that HP had stronger ability of forming complex with PF4. The difference of IC50 in this study between HP and LMWH is basically the same as the results at a PF4 concentration of 0.125 µM determined with SPR, the IC50 of LMWH was 2.4 µg/ml–2.9 μg/ml and that of HP was 0.38 µg/ml–0.6 μg/ml, which further verified the reliability of the BLI method (Zhang et al., 2020).This result was consistent with the reported clinical statistics that the probability of HIT induced by LWMH reduced by 65% compared with HP (Arepally, 2017). The main difference between LMWH and HP is their sulfation patterns and molecular sizes. The average molecular weight of HP and LMWH is 16 kDa and 4.5 kDa, respectively. It has been reported that the binding modes of shorter HP chains and longer HP chains with PF4 were different. With the increase of polymerization degree, the HP chains could bind to more than one PF4, and the hydrophobic interaction between multiple PF4 also helped to stabilize the complex (Nguyen T. H. et al., 2015; Nguyen et al., 2020). In addition, the appropriate level of sulfate content in HP and LMWH was 1.9–2.4 mol/disaccharide and not less than 1.8 mol/disaccharide, respectively. Therefore, the immunogenicity of HP and LMWH drugs might also correlate to their sizes of backbone chains and sulfation patterns.

## Conclusion

HP is an anticoagulant drug that has a long history of clinic use for over 90 years. Although associated with the risk of HIT, the place of HP is unreplaceable. LWMHs are improved versions of HP with reduced side effects, but the risk of HIT still exits. The immunogenicity risk of HP or LMWH may be induced by the contamination of proteins or nucleic acids during extraction of starting materials from porcine intestine mucosa. However, the polysaccharide itself binding to PF4 is recognized as the main factor of triggering the immune system and cause HIT. The evaluation of immunogenicity for HP and LMWH has been required by many of drug surveillance organizations, such as the Food and Drug Administration of the United States, the European Medicines Agency and the Center for Drug Evaluation, China. Since the HIT is a very complicated process, it is necessary to characterize the complex formed between PF4 and HP or LMWH from multiple aspects, include the size, surface charge, binding affinity, and so on. Among these features, binding affinity is the most straight-forward aspect to reflect the tendency of HP or LMWH to form complex with PF4. The competitive BLI method developed herein offers a convenient approach to demonstrate the binding behavior of HP to PF4. It is also a sensitive way to compare different HP and LMWH products, which can be integrated to the package of methodologies for ensuring the drug safety of HP and LMWH products.

## Data Availability

The original contributions presented in the study are included in the article/Spplementary Material, further inquiries can be directed to the corresponding authors.
