# Supplementary material for: Evaluating the immunogenicity of heparin and heparin derivatives by measuring their binding to platelet factor 4 using biolayer interferometry
Source: Front Mol Biosci. 2022 Aug 26;9:966754. doi: 10.3389/fmolb.2022.966754 (PMC9458964; doi:10.3389/fmolb.2022.966754)
Supplement: Supplementary file 1 [file DataSheet1.docx]

Supplementary Material

## Supplementary Figures


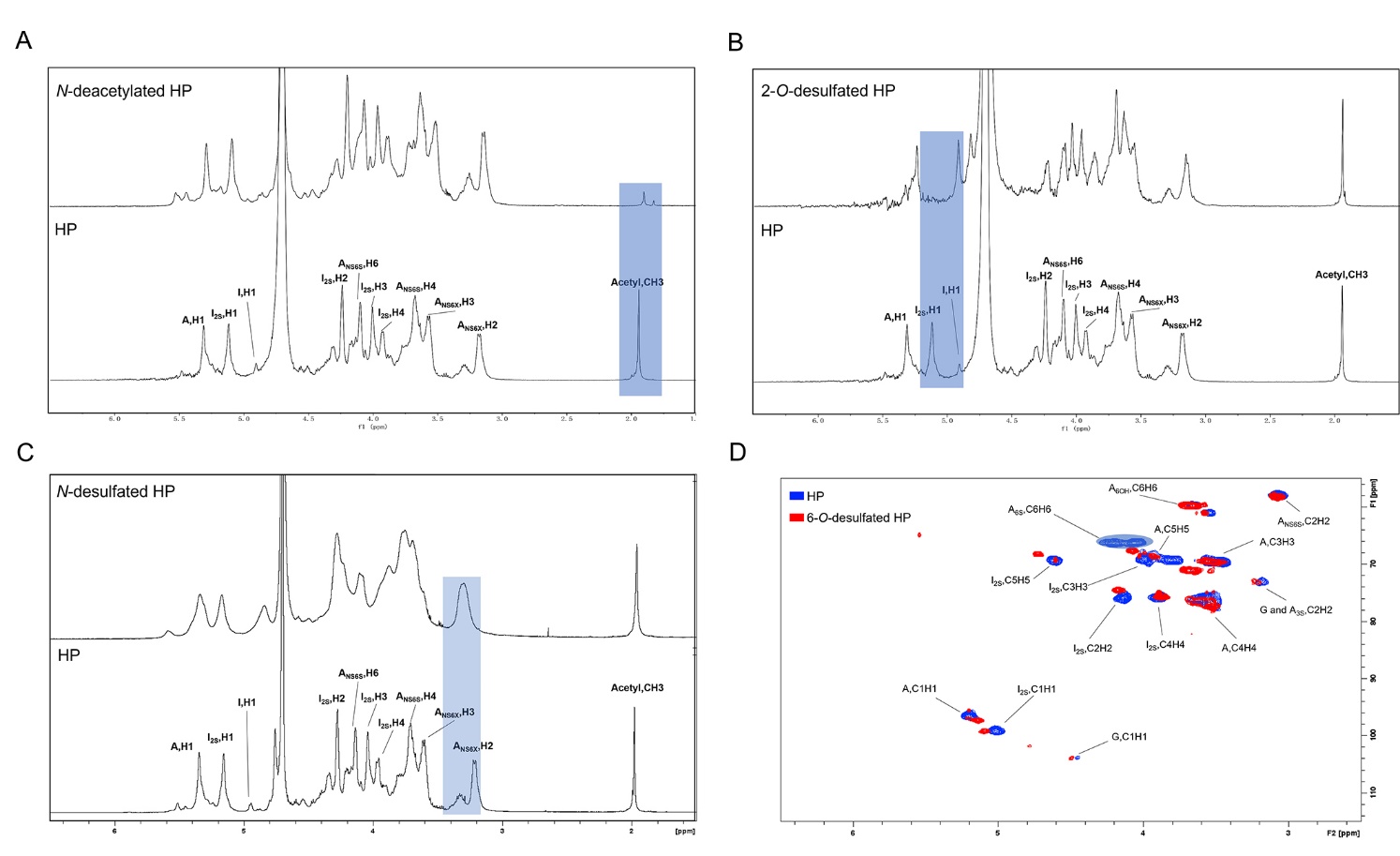


**Supplementary Figure S1.** Structural characterization and purity determination of HP derivatives. (A) The overlaid ^1^H-NMR spectra of *N*-deacetylated HP and HP. (B) The overlaid 1H-NMR spectra of 2-*O*-desulfated HP and HP. (C) The overlaid ^1^H NMR spectra of *N*-desulfated HP and HP. (D) The overlaid ^1^ H-^13^C HSQC NMR spectra of 6-*O*-desulfated HP (red) and HP (blue).
